# Surface-associated *Candida auris* recovery during five intensive care unit cluster responses: a retrospective descriptive study with structured evidence mapping

**DOI:** 10.3389/fcimb.2026.1955493

**Published:** 2026-09-29

**Authors:** Hui Wang, Kai Zhang, Tianfen Liao, Hongrong Cao, Pingping Wang

**Affiliations:** 1 Healthcare-Associated Infection Control Center, Sichuan Academy of Medical Sciences, Sichuan Provincial People’s Hospital, School of Medicine, University of Electronic Science and Technology of China, Chengdu, China; 2 Department of Laboratory Medicine, Sichuan Academy of Medical Sciences, Sichuan Provincial People’s Hospital, School of Medicine, University of Electronic Science and Technology of China, Chengdu, China

**Keywords:** *Candida auris*, environmental sampling, evidence mapping, healthcare surfaces, infection prevention and control, reusable medical equipment, surface disinfection, surface-associated recovery

## Abstract

**Background:**

Surface-associated persistence of *Candida auris* can complicate infection prevention in healthcare settings, particularly when reusable devices and mobile care items move across patient-care areas. During cluster responses, targeted environmental sampling may help identify surface and equipment categories for infection-prevention review.

**Methods:**

We retrospectively reviewed environmental culture records collected during five *C. auris* cluster responses in the surgical and medical intensive care units of Sichuan Provincial People’s Hospital between August 2024 and May 2026. Patient, healthcare-worker, liquid, and product samples were excluded. Environmental site names were standardized using a 10-category functional framework based on item function, patient proximity, mobility or sharing, and cleaning responsibility. A secondary structured evidence-mapping search of PubMed, Web of Science Core Collection, Embase (Elsevier), and Scopus assessed whether locally identified surface and equipment categories had been reported in previous healthcare investigations.

**Results:**

Among 177 environmental sampling records, 9 were culture-positive for *C. auris* (record-level culture yield, 5.1%). Positive records involved six standardized sites and five environmental categories. SpO_2_ probes/clips accounted for four positive records and were the only standardized site type recovered in more than one cluster response. Other positive sites were a bed rail/bed unit, transfer board, chlorhexidine measuring cup, soiled-linen/textile cart, and hair-washing/bathing equipment. Each locally positive category was represented in the published evidence map.

**Conclusion:**

*C. auris* was recovered from both fixed near-patient surfaces and reusable or mobile healthcare items, including monitoring accessories, transfer equipment, care-related items, and linen-handling equipment. These observations identify surface and equipment categories for consideration during infection-prevention review, particularly in relation to between-patient disinfection, drying, storage, equipment assignment, and separation of clean and used items. Because sampling was purposive and isolate relatedness was not assessed, these findings cannot be used to estimate environmental contamination prevalence, establish transmission, or evaluate intervention effectiveness.

## 1 Introduction

*Candida auris*, also referred to as *Candidozyma auris* in recent taxonomic literature, is an emerging healthcare-associated yeast characterized by persistent colonization, invasive infection, and difficult-to-control transmission in healthcare settings (Eyre et al., 2018; World Health Organization, 2022; Centers for Disease Control and Prevention, 2026). Compared with many clinically encountered *Candida* species, *C. auris* is more difficult to identify reliably in routine laboratories, can persist on healthcare surfaces, and may recontaminate patient-care environments after cleaning and disinfection (Keighley et al., 2021; Sansom et al., 2024). Patients in intensive care units (ICUs) are at increased risk because of severe underlying illness, invasive procedures, and prolonged or repeated antimicrobial exposure (Shastri et al., 2020; Briano et al., 2022).

Environmental contamination remains a major challenge in preventing healthcare-associated transmission of *Candida auris* (Eyre et al., 2018; Sansom et al., 2024). Previous investigations have recovered *C. auris* from a broad range of healthcare surfaces and equipment, including high-touch near-patient surfaces, bed units, monitoring equipment, reusable medical accessories, mobile equipment, care-related items, textiles, and wet-area surfaces (Eyre et al., 2018; Escandón et al., 2019; Yadav et al., 2021). Persistence on these inanimate surfaces may sustain environmental contamination and create opportunities for indirect transfer when cleaning, disinfection, drying, storage, or equipment-sharing practices are suboptimal (Ku et al., 2018; Dire et al., 2023). During suspected outbreaks or clusters, risk-based environmental surveillance can identify surface and device categories requiring immediate infection-prevention review (Tsay et al., 2018; Centers for Disease Control and Prevention, 2024).

Antimicrobial surface modifications and treated healthcare textiles have been investigated as complementary environmental infection-prevention approaches (Schmidt et al., 2012; Adlhart et al., 2018; Schneider et al., 2023). The potential contribution of these approaches is best considered alongside real-world evidence on where *C. auris* is recovered within healthcare environments. However, environmental investigations of *C. auris* differ substantially in sampling targets, sampling timing, detection methods, site nomenclature, and reporting formats (Leach et al., 2018; Silva et al., 2024). Some studies report only the total number of environmental samples and individual positive items, whereas others use molecular methods to investigate relationships among patient, environmental, and healthcare-worker isolates (Adams et al., 2018; Escandón et al., 2019). This heterogeneity limits the translation of environmental findings into reproducible priorities for surveillance, cleaning, disinfection, and equipment management.

Building on previous reports of *C. auris* recovery from healthcare surfaces and shared equipment, the present study focused on management-relevant surface and device types identified across repeated ICU cluster responses. We retrospectively characterized surface-associated *C. auris* recovery during five ICU cluster responses, standardized the recorded environmental site names using a functional classification, and contextualized the locally positive categories through structured evidence mapping. The study question was descriptive: which healthcare surface and equipment types yielded *C. auris* in environmental culture records generated during these cluster responses, and were the corresponding functional categories also represented in published healthcare environmental investigations? The study was not designed to estimate environmental contamination prevalence, compare contamination risks among environmental categories, evaluate intervention effectiveness, or reconstruct patient-level transmission.

## 2 Materials and methods

### 2.1 Study design and setting

We conducted a single-center retrospective descriptive study of environmental investigations performed during five ICU *C. auris* cluster responses, supplemented by a structured evidence map. The primary analysis was restricted to environmental culture records collected during these responses. The evidence-mapping component was used to determine whether the environmental site categories identified locally had previously been reported in healthcare investigations; it was not designed to estimate pooled contamination frequencies or intervention effects.

Data were obtained from five cluster responses conducted in the surgical and medical intensive care units of Sichuan Provincial People’s Hospital between August 2024 and May 2026. The primary analytical dataset comprised environmental sampling records. Aggregate, de-identified patient information was used only to describe the circumstances that triggered each response; identifiable patient information, individual-level clinical outcomes, and patient-level transmission relationships were not analyzed. Samples collected from patients or healthcare workers, as well as suction water, coupling gel, disinfectants, and other liquid or product samples, were excluded.

During the study period, the surgical and medical ICUs had 36 and 19 beds, respectively. During the cluster responses, environmental sampling focused primarily on case-associated bed areas, high-touch near-patient surfaces, reusable monitoring accessories, mobile equipment, transfer items, and reusable items used during patient care or cleaning.

### 2.2 Definition and inclusion of cluster responses

For retrospective response selection, an eligible cluster response was defined as an event in which at least three patients with *C. auris* colonization or infection were identified in the same ICU within a 7-day period and the infection prevention and control team initiated an enhanced response, including environmental sampling, because healthcare-associated transmission could not be excluded. A *C. auris*-positive patient was defined as a patient with *C. auris* documented in a clinical or surveillance specimen in contemporaneous records. Infection versus colonization was assigned as recorded by the infection prevention and control team and was not re-adjudicated for this study. This study-specific operational definition was applied retrospectively to identify eligible responses. It was informed by the investigation principles described in WS/T 524—2025, Standard for the Control of Healthcare-Associated Infection Outbreaks (National Health Commission of the People’s Republic of China, 2025), but did not reproduce a formal outbreak definition.

The operational definition identified events that triggered environmental sampling and infection prevention activities; it did not confirm an outbreak, ICU acquisition, or a shared transmission chain. Because some patients were colonized rather than infected, some had tested positive before ICU admission, and molecular relatedness was not assessed among patient isolates or between patient and environmental isolates, the five events are referred to as cluster responses rather than confirmed outbreaks. Aggregate epidemiological information available at the initiation of each response is summarized in **Supplementary Table S1**.

### 2.3 Data sources and unit of analysis

Source data were obtained from environmental sampling result forms retained by the Healthcare-Associated Infection Control Center, the participating ICUs, and the microbiology laboratory. The extracted variables included response period, ICU type, sampling date, de-identified environmental site name, standardized site name, site category, composite-record status, culture result, and positivity status.

The unit of analysis was one laboratory result linked to one environmental sampling record. When an original record combined two or more sampled surfaces, it was retained as one composite environmental sampling record and was not split into separate component records. Composite records were retained in the overall and response-specific denominators. A composite record was included once in a category-specific denominator only when all component surfaces mapped to the same environmental category. Composite records spanning multiple or indeterminate categories were excluded from category-specific denominators. Composite records were not used for standardized-site-specific denominator calculations because the laboratory result could not be attributed to an individual component surface. When the same standardized site was sampled separately at different beds, on different devices, or on different dates, each laboratory result was counted as a separate environmental sampling record.

### 2.4 Environmental surface sampling strategy

Environmental surface sampling was conducted during each cluster response by dedicated infection prevention and control staff under institutional procedures based on GB 15982—2012, Hygienic Standard for Disinfection in Hospitals, and local standard operating procedures (General Administration of Quality Supervision, Inspection and Quarantine of the People’s Republic of China, Standardization Administration of China, 2012). The institutional procedure specified the sampling area and general swabbing approach according to surface size and geometry. When the sampled surface area was <100 cm², the entire surface was sampled. When the area was ≥100 cm², a 100 cm² surface area was sampled. For flat surfaces, a sterile template was used to define the sampling area, and sterile swabs moistened with sampling solution were moved horizontally and vertically across the area. For small or irregular surfaces, such as peripheral oxygen saturation (SpO_2_) probes/clips and door handles, the surface was swabbed directly. When residual disinfectant was likely to be present, an appropriate neutralizer was added to the sampling solution.

Environmental sampling was purposive and guided by the field risk assessment conducted during each cluster response. It was not conducted as random sampling, fixed-site surveillance, or a prespecified before–after cleaning assessment. Sites were selected according to case-bed distribution, opportunities for patient or healthcare-worker contact, shared-equipment use, item reusability, frequency of handling, difficulty of cleaning and disinfection, and on-site risk assessment. Final sampling sites were determined through multidisciplinary discussion among infection prevention professionals, intensive care unit clinical staff, and microbiology laboratory staff. The discussion considered patient-care workflows, equipment sharing, environmental contact opportunities, and the feasibility of microbiological sampling. Accordingly, the sampling scope reflected the field risk assessment for each response rather than exhaustive environmental surveillance. Sampling times were not standardized across responses, and environmental sampling was not scheduled in relation to routine cleaning or disinfection. Under routine ICU practice during the study period, environmental surfaces were disinfected twice daily using a chlorine-containing disinfectant at 500 mg/L available chlorine, whereas medical instruments and equipment were wiped twice daily with 75% alcohol. However, the interval between the most recent cleaning or disinfection and individual sampling events was not consistently documented; therefore, the cleaning or disinfection status of a specific sampled surface at the time of sampling could not be determined reliably. The specific sampling solution and neutralizer formulation were also not consistently documented in the retrospective records. Patient-assignment status (patient-dedicated versus shared) was not consistently documented for individual reusable devices at the time of sampling. Post-cleaning resampling was not performed according to a standardized, prespecified protocol across the five cluster responses; therefore, the available environmental records could not be used to evaluate changes in culture results after cleaning or cleaning effectiveness. Accordingly, a negative culture result was not interpreted as evidence that an individual site or environmental category was uncontaminated.

### 2.5 Microbiological culture, identification, and positivity definition

Environmental surface swabs were transported to the microbiology laboratory within 4 h of collection. Samples were inoculated onto Sabouraud agar and incubated aerobically at 36 ± 1 °C for 48 h. Enrichment culture and direct molecular testing were not performed.

Yeast-like colonies were identified using the VITEK MS matrix-assisted laser desorption/ionization time-of-flight mass spectrometry system (bioMérieux, Marcy-l’Étoile, France; Knowledge Base v3.3). For this retrospective study, a culture-positive environmental record was defined as a record for which the routine VITEK MS report identified *C. auris* as the single species-level result. Reports classified as low discrimination, no identification, or bad spectrum were not considered *C. auris*-positive. No study-specific validation of VITEK MS Knowledge Base v3.3 was performed for *C. auris* isolates recovered from environmental surfaces. The original spectra were not reanalyzed as part of this retrospective study, and species identification was not confirmed by sequencing or an independent identification platform.

Whole-genome sequencing or other strain typing was not performed to assess relatedness between patient and environmental isolates. Accordingly, environmental recovery was not interpreted as evidence of a transmission source, transmission direction, or shared transmission chain.

### 2.6 Standardization and functional classification of healthcare surfaces and devices

To facilitate descriptive organization across cluster responses and contextualization with published studies, original local environmental site names were first standardized. Based on the complete standardized local site inventory, a 10-category operational framework was developed retrospectively after the cluster responses and finalized before literature data extraction. The investigators were aware of the environmental culture results during framework development and local site coding. The framework considered four attributes: item function, proximity to the patient, mobility or sharing between bed spaces, and cleaning responsibility. It was developed for the purposes of the present study and was not intended as a validated environmental risk-classification system.

The framework was used to describe management-relevant attributes of sampled items, including patient proximity, mobility or sharing, reusability, and cleaning responsibility. It was not intended to rank environmental categories by contamination risk.

The categories were: (1) cleaning/care-related reusable items; (2) environmental infrastructure and general surfaces; (3) mobile equipment, vehicles, and transfer items; (4) other treatment and life-support equipment; (5) patient monitoring and diagnostic equipment; (6) patient near-zone environmental surfaces; (7) respiratory and airway-related equipment; (8) textile, curtain, and waste-related items; (9) water sources, wet areas, and living facilities; and (10) workstation and shared high-touch equipment. Two investigators, neither of whom was blinded to the environmental culture results, independently assigned each original site name to a standardized site, subcategory, and category using the finalized framework. Disagreements were resolved by consensus and, when necessary, adjudication by a third investigator. The operational category definitions and boundary rules are provided in **Supplementary Table S2A**, and the site-level coding and mapping decisions are provided in **Supplementary Table S2B**.

### 2.7 Outcomes

The primary outcomes were the overall and response-specific numbers of environmental sampling records and culture-positive records. Record-level culture yield was calculated as the number of culture-positive environmental records divided by the total number of environmental sampling records collected during the corresponding response.

Secondary outcomes were the standardized sites and categories represented among culture-positive records, the numbers of eligible environmental records within locally positive categories, and standardized site types with culture-positive records in more than one response.

### 2.8 Statistical analysis

Analyses were descriptive. Counts and record-level percentages were reported for the overall dataset and individual cluster responses. Because environmental sampling was purposive and differed across responses in site selection, sampling time, sample size, and cleaning or disinfection context, no confidence intervals or hypothesis tests were calculated. Inferential comparisons were not prespecified because the purposive sampling design did not provide a common probability-based sampling frame across cluster responses or environmental categories. Record-level percentages describe only the yield of the sampled records and were not interpreted as estimates of environmental contamination prevalence or used for direct comparisons between responses or site categories. Data management and descriptive analyses were performed using Microsoft Excel 2021.

### 2.9 Secondary structured evidence mapping

A secondary structured evidence-mapping component was conducted to determine whether the healthcare surface and device categories identified locally had previously been reported as *C. auris*-positive in healthcare settings. The evidence map was intended to contextualize the local findings and describe which environmental categories had also been reported as *C. auris*-positive in previous healthcare investigations. It was not designed to estimate pooled positivity, compare contamination risks among categories, or evaluate intervention effectiveness.

PubMed, Web of Science Core Collection, Embase (Elsevier), and Scopus were searched from database inception to July 5, 2026. Search terms covered *Candida auris* or *Candidozyma auris*, environmental sampling or contamination, healthcare settings, medical equipment, reusable care items, and healthcare environments. Search syntax was adapted for each database. No language restrictions were applied at the search or study-selection stages. No separate search of gray literature or institutional websites was conducted. The complete search strategies and numbers of records retrieved from each database are provided in **Supplementary Table S3**.

Studies were eligible if they met all of the following criteria: (1) the setting was a hospital, intensive care unit, long-term care facility, skilled nursing facility, or another institution providing medical or nursing care; (2) the study involved *C. auris* infection, colonization, clustering, an outbreak investigation, or another targeted healthcare environmental investigation; (3) testing was performed on inanimate healthcare surfaces, medical equipment, reusable care items, or ventilation-system surfaces; and (4) at least one *C. auris*-specific environmental result was extractable. Extractable results included an environmental numerator and denominator, a clearly reported entirely negative environmental investigation, or at least one specifically identified positive environmental site. Studies were not required to report a positive environmental result. Eligible publication types included original research articles, outbreak or cluster investigation reports, public health investigation reports, and brief reports or communications containing original environmental data. Studies using culture-based, molecular, or combined detection methods were eligible.

Studies were excluded if they involved only non-healthcare environments; reported only patient or healthcare-worker screening; tested only air, water, wastewater, medical solutions, disinfectants, coupling gel, or other liquid products without eligible inanimate-surface data; did not provide a *C. auris*-specific environmental result distinguishable from findings for other organisms; were limited to simulated contamination, *in vitro* survival, or disinfectant-efficacy experiments; or were reviews, editorials, guidelines, conference abstracts without sufficient original data, or duplicate or overlapping reports without additional eligible environmental information. Passive air samples were excluded from surface-category mapping, whereas swabs collected from ventilation grilles, filters, air-handling components, or other inanimate ventilation-system surfaces were eligible.

Retrieved records were imported into EndNote 20 (Clarivate, Philadelphia, PA, United States). Duplicate records were identified using the EndNote duplicate-detection function and were subsequently verified using titles, authors, publication years, and digital object identifiers. Two investigators independently screened titles and abstracts and subsequently assessed potentially eligible full-text reports. Disagreements regarding study eligibility were resolved through discussion and, when necessary, adjudication by a third investigator. Reasons for full-text exclusion were recorded, and the study-selection process was summarized in a flow diagram.

Two investigators independently extracted the data and assigned the reported environmental sites to the 10-category framework. Disagreements were resolved by consensus and, when necessary, adjudication by a third investigator. Extracted variables included author, publication year, country or region, healthcare setting, event type, number of affected or colonized patients, environmental sampling design and timing, environmental numerator and denominator, positive environmental sites, detection method, patient–environment relatedness analysis, cleaning or disinfection measures, and reported limitations. The positivity status of each environmental investigation, the availability of specific positive-site information, and the completeness of the environmental numerator and denominator were also recorded. Environmental site names reported in the included studies were mapped to the same 10-category framework used for the local dataset.

Detection method and quantitative reporting completeness were classified as separate dimensions. Detection evidence was classified at the study–category level as follows: (1) culture-based evidence, when viable *C. auris* was recovered or an environmental site within the category was explicitly reported as culture-positive; (2) molecular-only evidence, when *C. auris* DNA was detected without culture confirmation; or (3) mixed or method-undifferentiated evidence, when both culture and molecular methods were used but the detection method could not be assigned to the reported environmental site or item group. Detection classes were assigned separately to each environmental category reported within a study. Consequently, a single study could contribute different detection classes across environmental categories when the reported detection methods differed by site or when site-specific methods could not be distinguished.

Quantitative reporting completeness was classified as follows: (1) a complete binary environmental numerator and denominator; (2) one or more specific positive environmental sites without a complete denominator; (3) quantitative culture-burden evidence reported as colony counts rather than binary positivity; or (4) an eligible entirely negative environmental investigation without a complete denominator.

For category-level evidence mapping, each study was counted no more than once within each environmental category, regardless of the number of positive sites, sampling rounds, or sampling time points reported. When both culture-based and molecular-only evidence were available for the same study and category, the study was counted in the culture-based group only. Eligible entirely negative studies, passive air samples, and reports of aggregate environmental positivity without an identifiable positive subsite did not contribute to category-level positive findings. No pooled environmental positivity estimate or meta-analysis was undertaken because of substantial heterogeneity in sampling targets, sampling times, cleaning contexts, detection methods, and reporting completeness. No formal risk-of-bias assessment was performed because the evidence map was used for descriptive category-level contextualization rather than comparative risk estimation or intervention-effect assessment.

### 2.10 Ethics and privacy protection

This study was reviewed by the Ethics Committee of Sichuan Provincial People’s Hospital and granted an exemption from full ethical review (approval no. 2025-385). The analysis used previously collected, de-identified environmental microbiology records and aggregate, non-identifiable case information solely to describe the context of each cluster response. No human specimens, identifiable patient information, or additional interventions were included. The requirement for informed consent was waived by the Ethics Committee.

## 3 Results

### 3.1 Local analytical dataset and literature screening results

The local dataset comprised environmental culture records collected during five ICU *C. auris* cluster responses conducted between August 2024 and May 2026, including four in the surgical ICU and one in the medical ICU. All five responses met the study-specific eligibility criterion of at least three patients with *C. auris* colonization or infection identified in the same ICU within a 7-day period. Aggregate patient and response characteristics at initiation are summarized in **Supplementary Table S1**, and the complete environmental sampling dataset is provided in **Supplementary Table S4**.

The database searches identified 892 records: 222 from PubMed, 208 from Web of Science Core Collection, 218 from Embase (Elsevier), and 244 from Scopus. After removal of 510 duplicates, 382 records underwent title and abstract screening, of which 330 were excluded. Fifty-two reports were sought for retrieval, and two could not be obtained. Fifty full-text reports were assessed; 32 studies were included and 18 were excluded. The search and study-selection process is shown in **Figure 1**.

**Figure 1:**
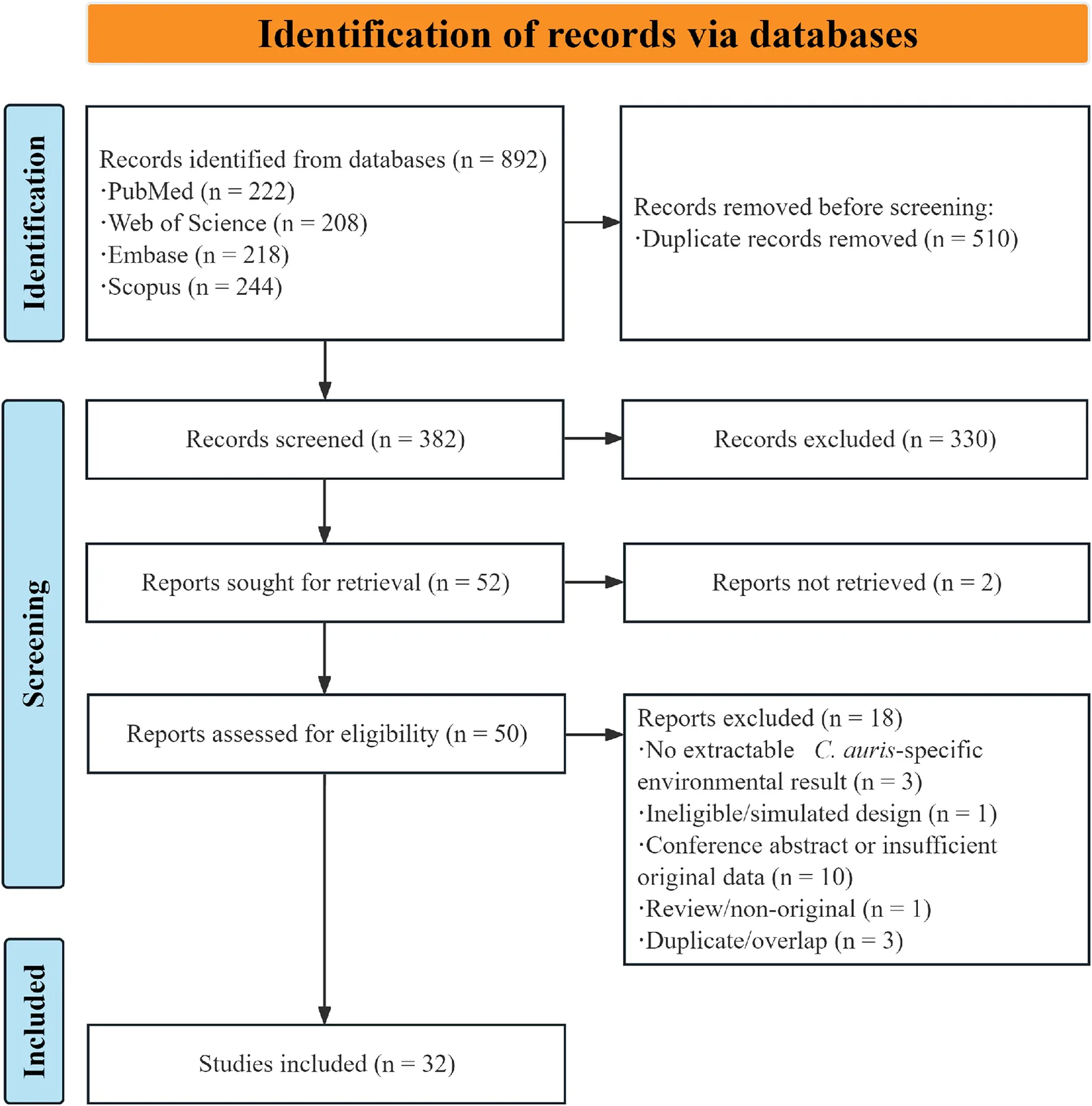
Flow diagram of the secondary structured evidence-mapping search and study selection. Records were identified from PubMed (n = 222), Web of Science Core Collection (n = 208), Embase (Elsevier) (n = 218), and Scopus (n = 244), yielding 892 records before deduplication. After removal of 510 duplicates, 382 records underwent title and abstract screening, of which 330 were excluded. Fifty-two reports were sought for retrieval; two could not be obtained, and 50 full-text reports were assessed. Thirty-two studies were included, and 18 full-text reports were excluded: no extractable *C. auris*-specific environmental result (n = 3), ineligible or simulated design (n = 1), conference abstract or insufficient original data (n = 10), review or non-original publication (n = 1), and duplicate or overlapping report (n = 3).

### 3.2 Environmental culture results from five ICU cluster responses

Across the five cluster responses, *C. auris* was recovered from 9 of 177 environmental sampling records (record-level culture yield, 5.1%). Culture-positive records were identified during the August 2024 surgical ICU, April 2025 surgical ICU, and May 2026 medical ICU responses. No culture-positive environmental records were identified during the other two surgical ICU responses. Response-specific sampling volumes and culture results are presented in **Table 1**. The timing of the five responses and their standardized culture-positive environmental sites are shown in **Figure 2**.

**Table 1:** Environmental culture results from five intensive care unit *Candida auris* cluster responses.

Response period	ICU type	Environmental sampling days, n	Environmental sampling records, n	Culture-positive records, n/N (%)
2024-08	Surgical ICU	4	55	4/55 (7.3)
2025-04	Surgical ICU	1	28	4/28 (14.3)
2025-08	Surgical ICU	1	30	0/30 (0.0)
2025-12	Surgical ICU	1	46	0/46 (0.0)
2026-05	Medical ICU	1	18	1/18 (5.6)
**Total**	**Surgical and medical ICUs**	**8**	**177**	**9/177 (5.1)**

ICU, intensive care unit. Record-level culture yield was calculated as the number of environmental sampling records that were culture-positive for *C. auris* divided by the total number of environmental sampling records collected during the corresponding response. Because sampling was purposive and differed across responses in site selection, timing, and cleaning context, these percentages describe only the yield of the sampled records and should not be interpreted as estimates of environmental contamination prevalence or used for direct comparisons between responses. Bold values indicate overall totals.

**Figure 2:**
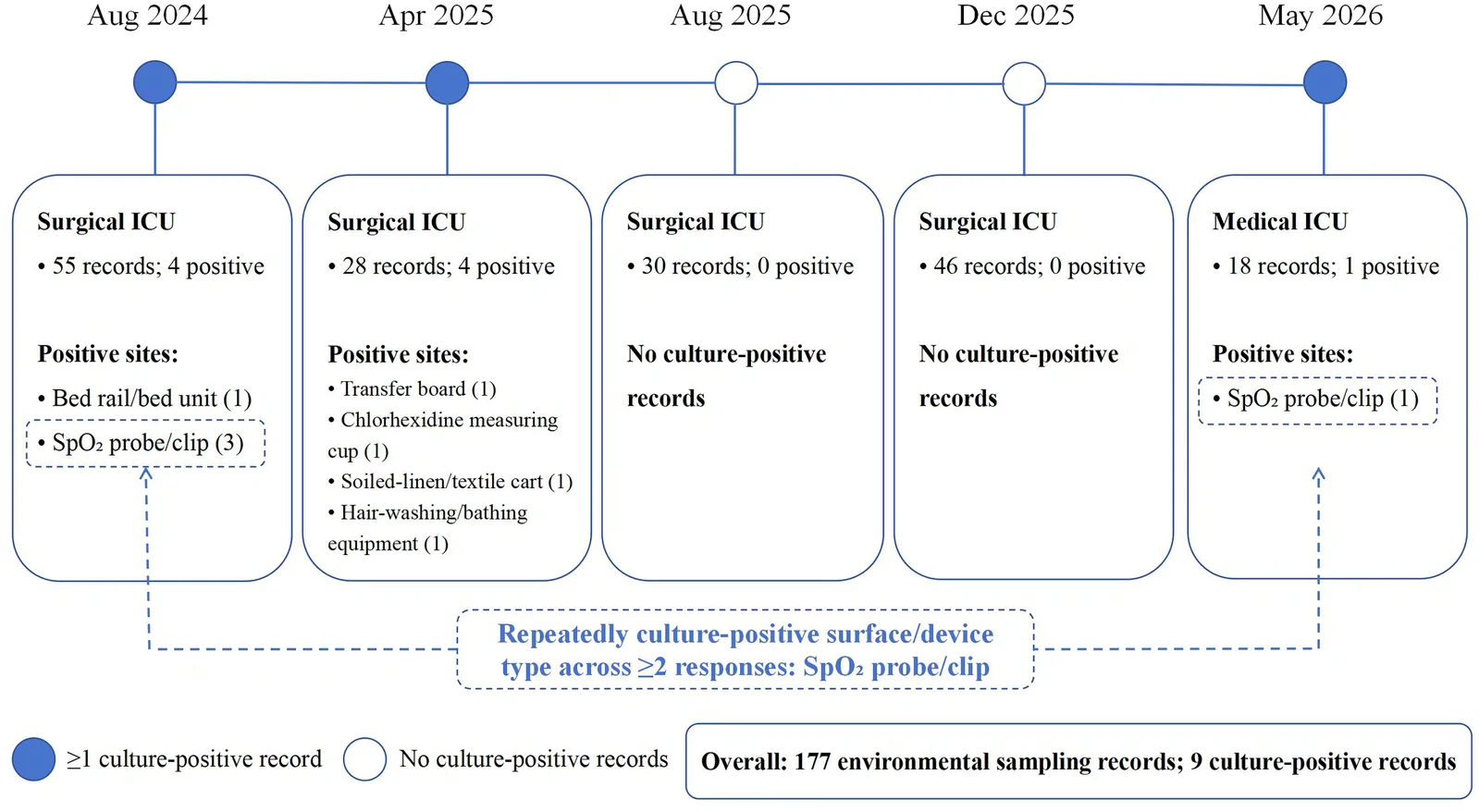
Timeline and environmental culture findings from five intensive care unit *Candida auris* cluster responses. Each time point represents one cluster response conducted between August 2024 and May 2026. Filled circles indicate responses with at least one culture-positive environmental record, whereas open circles indicate responses with no culture-positive environmental records. Boxes show the ICU type, number of environmental sampling records, number of culture-positive records, and standardized culture-positive sites identified during each response. Dashed arrows identify the SpO_2_ probe/clip as the only standardized site type with culture-positive records in more than one response. The bottom summary shows the overall numbers of environmental sampling records and culture-positive records across the five responses. ICU, intensive care unit; SpO_2_, peripheral oxygen saturation.

### 3.3 Surface-associated *C. auris* recovery across healthcare items

Across the nine culture-positive environmental records, *C. auris* was recovered from six standardized healthcare surface or equipment types: bed rail/bed unit, SpO_2_ probe/clip, transfer board, chlorhexidine measuring cup, soiled-linen/textile cart, and hair-washing/bathing equipment. Details of the individual culture-positive records are presented in **Table 2**.

**Table 2:** *Candida auris*-positive environmental records recovered during five intensive care unit cluster responses.

Cluster response	Sampling date	ICU type	Standardized positive site	Site category
SICU-2024-08	2024-08-14	Surgical ICU	Bed rail/bed unit	Patient near-zone environmental surfaces
SICU-2024-08	2024-08-14	Surgical ICU	SpO_2_ probe/clip	Patient monitoring and diagnostic equipment
SICU-2024-08	2024-08-14	Surgical ICU	SpO_2_ probe/clip	Patient monitoring and diagnostic equipment
SICU-2024-08	2024-08-20	Surgical ICU	SpO_2_ probe/clip	Patient monitoring and diagnostic equipment
SICU-2025-04	2025-04-17	Surgical ICU	Transfer board	Mobile equipment, vehicles, and transfer items
SICU-2025-04	2025-04-17	Surgical ICU	Chlorhexidine measuring cup	Cleaning/care-related reusable items
SICU-2025-04	2025-04-17	Surgical ICU	Soiled-linen/textile cart	Textile, curtain, and waste-related items
SICU-2025-04	2025-04-17	Surgical ICU	Hair-washing/bathing equipment	Cleaning/care-related reusable items
MICU-2026-05	2026-05-26	Medical ICU	SpO_2_ probe/clip	Patient monitoring and diagnostic equipment

ICU, intensive care unit; SICU, surgical intensive care unit; MICU, medical intensive care unit; SpO_2_, peripheral oxygen saturation. Culture positivity indicates recovery of *C. auris* from the corresponding environmental surface and does not establish that surface as a transmission source or route. SpO_2_ probe/clip was the only standardized site type with culture-positive records in more than one cluster response. Multiple rows with the same standardized site represent separate laboratory sampling records.

SpO_2_ probes/clips accounted for four of the nine culture-positive records and were the only standardized site type with culture-positive records in more than one response. Three culture-positive SpO_2_ probe/clip records were identified during the August 2024 surgical ICU response and one during the May 2026 medical ICU response. Each of the remaining five standardized sites accounted for one culture-positive record.

### 3.4 Distribution of positive records by surface and device category

The nine culture-positive records mapped to five environmental categories. Patient monitoring and diagnostic equipment accounted for four positive records, all involving SpO_2_ probes/clips. Cleaning/care-related reusable items accounted for two positive records, involving a chlorhexidine measuring cup and hair-washing/bathing equipment. Patient near-zone environmental surfaces; mobile equipment, vehicles, and transfer items; and textile, curtain, and waste-related items each accounted for one culture-positive record.

Of the 177 environmental sampling records, 169 were eligible for category-specific denominator calculations. Eight composite records spanning more than one environmental category were excluded from category-specific denominators but retained in the overall denominator. None of the eight cross-category composite records was culture-positive. Among eligible records, patient monitoring and diagnostic equipment comprised 33 records, cleaning/care-related reusable items 13, patient near-zone environmental surfaces 16, mobile equipment, vehicles, and transfer items 14, and textile, curtain, and waste-related items six. These counts describe the distribution of sampled records and were not used to compare contamination risks between categories.

### 3.5 Mapping of locally positive surface and device categories to published evidence

Among the 32 included studies (Schelenz et al., 2016; Biswal et al., 2017; Adams et al., 2018; Eyre et al., 2018; Lesho et al., 2018; Al Maani et al., 2019; Escandón et al., 2019; Kumar et al., 2019; Ruiz-Gaitán et al., 2019; Alfouzan et al., 2020; Jung et al., 2020; Pacilli et al., 2020; Patterson et al., 2021; Salah et al., 2021; Yadav et al., 2021; Mulet Bayona et al., 2022; Taori et al., 2022; Didik et al., 2023; McDougal et al., 2023; Wang et al., 2023; Anirima et al., 2024; Cerqueira et al., 2024; Di Lorenzo et al., 2024; Lee et al., 2024; Sansom et al., 2024; Xu et al., 2025; Aldoghaim et al., 2026; Fung et al., 2026; Tomé et al., 2026; Uğur et al., 2026; Vacca et al., 2026; Wong et al., 2026), 24 reported a complete binary environmental numerator and denominator, six reported one or more specific positive sites without a complete denominator, one reported quantitative culture-burden data rather than binary positivity, and one eligible investigation reported no positive environmental findings but did not provide a complete denominator.

Of the 32 included studies, 30 reported at least one eligible positive environmental finding, whereas two reported entirely negative eligible environmental investigations. One of the 30 positive studies reported aggregate environmental positivity without identifying the positive subsites and therefore did not contribute to category-level counts. The remaining 29 studies contributed a positive finding to at least one environmental category. Study-level characteristics are presented in **Supplementary Table S5**, study–category detection classes and counting decisions in **Supplementary Table S6**, and reasons for full-text exclusion in **Supplementary Table S7**.

Each of the five locally positive environmental categories was represented among the included studies. Culture-based, molecular-only, and mixed or method-undifferentiated study–category evidence counts are shown separately in **Figure 3**. The largest number of studies reporting positive environmental sites mapped to patient near-zone environmental surfaces (21 studies), followed by environmental infrastructure and general surfaces and patient monitoring and diagnostic equipment (16 studies each). These counts describe the distribution of reported evidence within the included studies and should not be interpreted as category-specific contamination frequencies, probabilities, or comparative risks. The overlap between local and published findings shows that the locally positive categories have also been reported in previous healthcare environmental investigations; these study counts do not imply differences in contamination risk between categories.

**Figure 3:**
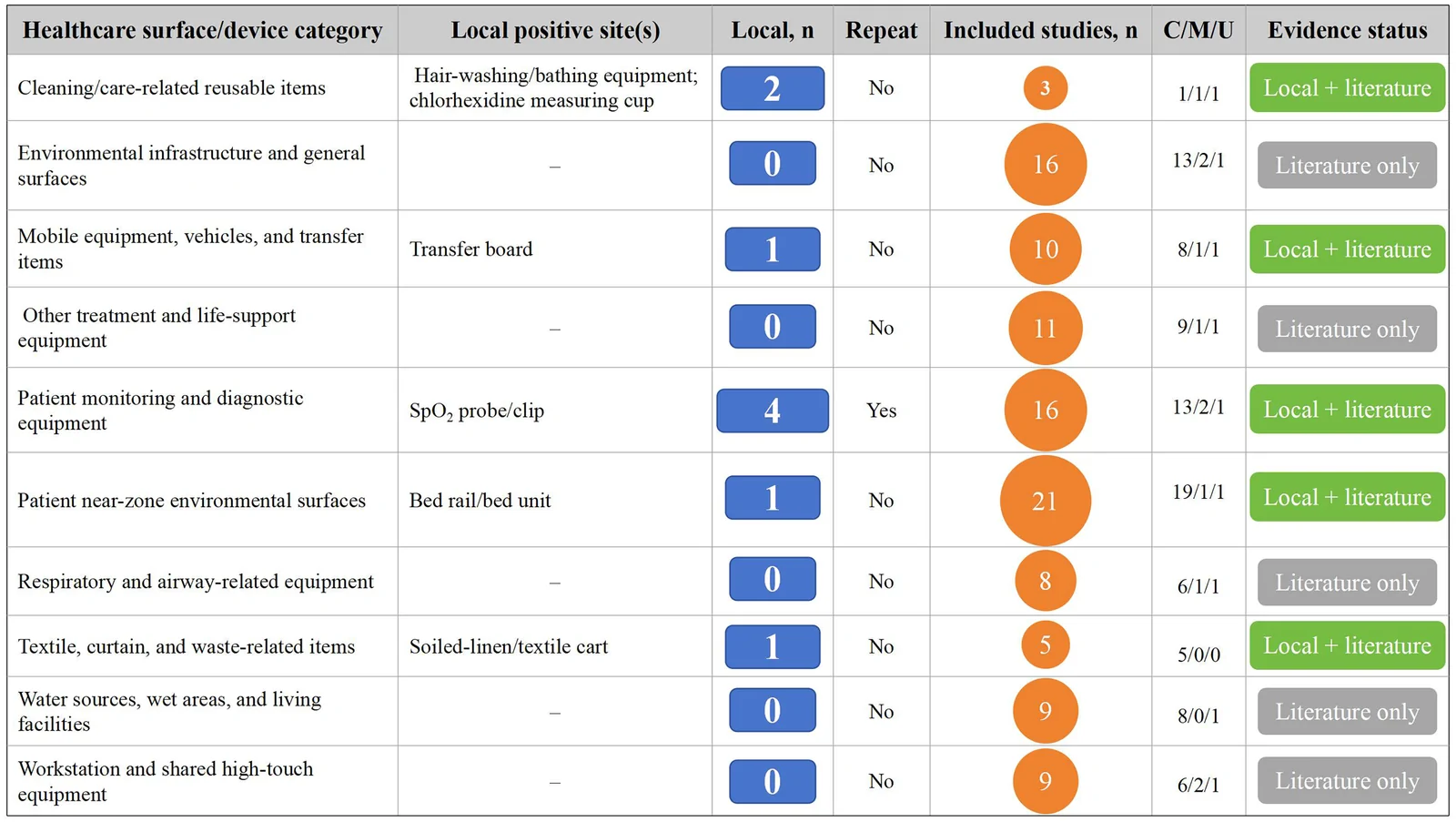
Surface-associated *Candida auris* evidence across healthcare environmental and device categories. “Local, n” denotes the number of local culture-positive environmental records. “Repeat” indicates whether a standardized site type was culture-positive in more than one cluster response. “Included studies, n” denotes the number of included studies reporting positive evidence within each category. Orange circle size scales with the number of included studies. C, M, and U indicate culture-based, molecular-only, and mixed or method-undifferentiated evidence, respectively. Each study was counted no more than once per category. Study counts indicate the extent of published reporting and do not represent category-specific contamination probability or comparative risk. “Local + literature” and “Literature only” indicate the source of evidence and do not represent evidence quality or comparative risk. SpO_2_, peripheral oxygen saturation.

## 4 Discussion

Across five ICU cluster responses, *C. auris* was recovered from both fixed near-patient surfaces and reusable or mobile items embedded in patient-care workflows. SpO_2_ probes/clips were the only standardized device type with positive records in more than one response, while additional positive records involved a transfer board, a chlorhexidine measuring cup, hair-washing/bathing equipment, and a soiled-linen/textile cart. Thus, the range of positive item types observed in these records extended beyond conventional fixed room surfaces. The functional classification and evidence map provide a descriptive framework for contextualizing these observations across published healthcare environmental investigations. The main contribution of this study is the structured identification of management-relevant surface and device types across repeated ICU cluster responses and their contextualization within a standardized functional evidence map.

*C. auris* was recovered from SpO_2_ probes/clips in two separate responses, making these accessories the only standardized site type positive in more than one response. Their frequent handling, direct patient contact, and potential movement between bed spaces make patient assignment, between-patient disinfection, labeling, and storage relevant processes for infection-prevention review. Previous investigations have similarly reported *C. auris* on reusable temperature probes, thermometers, blood pressure cuffs, and other monitoring or diagnostic accessories (Eyre et al., 2018; Anirima et al., 2024; Lee et al., 2024). During cluster responses, infection-prevention review may reasonably include patient or bed-space assignment, disinfectant compatibility and contact time, and separation of clean from used equipment (Centers for Disease Control and Prevention, 2024; UK Health Security Agency, 2025).

Patient near-zone surfaces remain relevant sampling targets during *C. auris* cluster responses. In the present study, *C. auris* was recovered from a bed rail/bed unit, and previous healthcare investigations have reported recovery from bed rails and other high-touch near-patient surfaces (Adams et al., 2018; Sansom et al., 2024). Frequent contact by patients, healthcare workers, and patient-care equipment creates multiple opportunities for contamination of these surfaces. This observation is consistent with the continued consideration of high-touch near-patient surfaces when risk-based environmental sampling is undertaken during *C. auris* cluster responses.

*C. auris* was also recovered from a transfer board. Because transfer items can move between beds, rooms, and clinical areas, post-use cleaning, disinfection, and equipment movement are relevant processes to consider during infection-prevention review. Previous investigations have reported *C. auris* on mobile carts, transfer equipment, and other shared patient-care devices (Pacilli et al., 2020; Anirima et al., 2024). Equipment movement and post-use cleaning or disinfection practices were not measured in this study; therefore, the transfer board finding should not be interpreted as evidence of a transmission pathway.

Recovery from hair-washing/bathing equipment, a chlorhexidine measuring cup, and a soiled-linen/textile cart extends the findings beyond conventional bedside surfaces. These items may be repeatedly used, handled during patient care, exposed to contaminated textiles or moisture, or moved between clinical areas. Previous investigations have reported *C. auris* on treatment carts, textiles, curtains, lanyards, and other movable or care-related items that may not be consistently included in routine bedside cleaning (Biswal et al., 2017; Adams et al., 2018; Patterson et al., 2021). Environmental review during cluster responses may therefore include reusable care items, drying and storage practices, and linen-handling and transport workflows.

No culture-positive records were identified locally from respiratory or life-support equipment, workstation or shared high-touch equipment, wet-area surfaces, or ventilation-related surfaces. However, published investigations have recovered *C. auris* from respiratory equipment, bathroom and other wet-area surfaces, shared equipment, and ventilation-system surfaces (Adams et al., 2018; Didik et al., 2023; Cerqueira et al., 2024). Local non-recovery should not be interpreted as evidence that these categories were uncontaminated because sampling scope, intensity, timing, and cleaning context varied across responses. Decisions to include these categories in future environmental sampling should be guided by case distribution, care workflows, equipment-sharing patterns, and site-specific risk assessment.

Differences in site terminology and reporting format limit direct comparison of specific culture-positive items across investigations. Mapping the local sites to functional categories enabled descriptive alignment with published evidence without implying that the frequency of reported categories represented contamination risk. The locally positive categories were also represented in the published evidence set, including patient monitoring equipment, near-patient surfaces, mobile or transfer items, reusable care items, and linen-related equipment. **Table 3** summarizes the local findings and corresponding infection-prevention processes for review.

**Table 3:** Surface and device categories with local *Candida auris* recovery and corresponding infection-prevention processes for review.

Surface/device category	Representative positive site(s)	Surface-management characteristic	IPC process for review
Patient monitoring and diagnostic equipment	SpO_2_ probe/clip	Small reusable accessory; direct patient contact; frequent handling; possible movement between beds	Patient assignment, between-patient disinfection, labeling, clean storage
Patient near-zone environmental surfaces	Bed rail/bed unit	Fixed high-touch surface with repeated hand and equipment contact	Daily and terminal cleaning
Mobile equipment, vehicles, and transfer items	Transfer board	Mobile shared item moving between beds and clinical areas	Post-use disinfection and clean/used separation
Cleaning/care-related reusable items	Hair-washing/bathing equipment; chlorhexidine measuring cup	Repeated reuse; moisture exposure; irregular geometry; drying and storage requirements	Cleaning, disinfection, complete drying, clean storage
Textile, curtain, and waste-related items	Soiled-linen/textile cart	Contact with contaminated textiles; movement across care areas	Linen workflow separation and cart decontamination

SpO_2_, peripheral oxygen saturation; IPC, infection prevention and control. Local culture-positive records were based on environmental sampling records collected during five intensive care unit cluster responses. Published category-level evidence is presented separately in **Figure 3**. The listed IPC processes were not assessed as exposure variables or evaluated for compliance in the local dataset. The listed processes identify areas for infection-prevention review and do not establish environmental sources, transmission routes, or patient–environment isolate relatedness.

Current infection prevention recommendations emphasize effective cleaning and disinfection of patient rooms, high-touch surfaces, and shared equipment during *C. auris* response activities (Centers for Disease Control and Prevention, 2024; Public Health Agency of Canada, 2024; UK Health Security Agency, 2025). The present observations are consistent with including small monitoring accessories, mobile transfer items, reusable care equipment, drying and storage practices, and linen-handling workflows in infection-prevention review. Future investigations would be strengthened by integrating environmental culture results with targeted patient screening based on epidemiological links or high-risk exposures (Centers for Disease Control and Prevention, 2025), bed-movement histories, equipment-use records, and molecular epidemiology. Future studies should assess how item design and reprocessing requirements affect cleaning performance under routine clinical conditions.

This study has several limitations. First, it was a single-center retrospective analysis of five ICU cluster responses, which limits generalizability. Environmental sampling was purposive and varied in scope, intensity, timing, and cleaning context; consequently, record-level culture yields cannot be interpreted as estimates of environmental contamination prevalence, compared directly across responses or categories, or used to assess cleaning effectiveness. Environmental sampling was not scheduled in relation to routine cleaning or disinfection. Although routine ICU practice during the study period consisted of twice-daily disinfection of environmental surfaces with a chlorine-containing disinfectant at 500 mg/L available chlorine and twice-daily wiping of medical instruments and equipment with 75% alcohol, the interval between the most recent cleaning or disinfection and individual sampling events was not consistently documented. Therefore, the cleaning or disinfection status of a specific sampled surface at the time of sampling could not be determined reliably. The specific sampling solution and neutralizer formulation were also not consistently documented, which limited procedural reproducibility and the interpretation of negative cultures. Some laboratory records combined multiple surfaces, preventing attribution of results to individual sites. Item-level information on material, design, age, and reprocessing was not consistently available.

Second, enrichment culture was not performed before direct inoculation onto Sabouraud agar. The absence of an enrichment step may have reduced recovery of low-burden or environmentally stressed *C. auris* cells and therefore may have lowered the observed record-level culture yield. The observed 5.1% represents the yield of the routine direct-culture approach used during these cluster responses rather than an estimate of the true frequency of environmental contamination. The magnitude of any under-detection cannot be quantified from the available retrospective data. Species identification relied on routine VITEK MS Knowledge Base v3.3 reporting. No study-specific validation was performed for environmental surface isolates, and identification was not confirmed by sequencing or an independent identification platform. Species-level misclassification therefore cannot be completely excluded.

Third, patient screening, bed-movement, and equipment-use data were not linked to environmental records. The retrospective dataset could not consistently determine whether individual reusable devices were patient-dedicated or shared at the time of sampling. Whole-genome sequencing or other strain typing was not performed to assess relatedness between patient and environmental isolates. Without isolate-relatedness data, environmental recovery cannot establish a transmission source, transmission direction, or shared transmission chain, nor determine the temporal relationship between patient colonization and environmental recovery.

Finally, the operational classification framework was developed retrospectively and has not been externally validated. The investigators who performed local site coding were not blinded to culture results; therefore, knowledge of positivity status may have influenced classification decisions and introduced classification or confirmation bias. Alternative classification rules could also yield different category assignments.

The evidence-mapping component also had limitations. It was not prospectively registered, did not include searches of gray literature or institutional websites, lacked a formal risk-of-bias assessment, and was limited by heterogeneity in sampling and reporting. It should therefore be interpreted as a descriptive map of reported environmental sites rather than an assessment of evidence strength or category-specific risk.

## 5 Conclusion

Retrospective review of environmental culture records from five ICU *C. auris* cluster responses documented recovery from fixed high-touch surfaces, reusable monitoring accessories, mobile transfer items, and care- and linen-handling equipment. SpO_2_ probes/clips were the only standardized site type recovered in more than one response. Together with the structured evidence map, these observations describe management-relevant surface and equipment categories that may be considered during infection-prevention review. Because environmental sampling was purposive and isolate relatedness was not assessed, these findings cannot be used to estimate environmental contamination prevalence, establish transmission, or demonstrate the effectiveness of an infection-prevention intervention.

## Data Availability

The original contributions presented in the study are included in the article/**Supplementary Material**. Further inquiries can be directed to the corresponding author.
