# Supplementary material for: Surface-associated Candida auris recovery during five intensive care unit cluster responses: a retrospective descriptive study with structured evidence mapping
Source: Front Cell Infect Microbiol. 2026 Sep 29;16:1955493. doi: 10.3389/fcimb.2026.1955493 (PMC13657941; doi:10.3389/fcimb.2026.1955493)
Supplement: Supplementary file 1 [file Table3.docx]

**Supplementary Table S3.** Detailed literature search strategy.

| **Database** | **Search strategy** | **Records retrieved** |
| --- | --- | --- |
| PubMed | (("*Candida auris*"[Mesh] OR "*Candida auris*"[Title/Abstract] OR "*C. auris*"[Title/Abstract] OR "*Candidozyma auris*"[Title/Abstract]) AND ("Environmental Monitoring"[Mesh] OR "Fomites"[Mesh] OR "environmental sampling"[Title/Abstract] OR "environmental surveillance"[Title/Abstract] OR "environmental culture*"[Title/Abstract] OR "environmental swab*"[Title/Abstract] OR "surface sampling"[Title/Abstract] OR "surface culture*"[Title/Abstract] OR "surface swab*"[Title/Abstract] OR "environmental contamination"[Title/Abstract] OR "surface contamination"[Title/Abstract] OR "healthcare environment*"[Title/Abstract] OR "health care environment*"[Title/Abstract] OR "hospital environment*"[Title/Abstract] OR fomite*[Title/Abstract] OR (environment*[Title/Abstract] AND (surface*[Title/Abstract] OR equipment[Title/Abstract] OR device*[Title/Abstract] OR "medical equipment"[Title/Abstract] OR "reusable equipment"[Title/Abstract] OR "patient room*"[Title/Abstract]))) AND ("Health Facilities"[Mesh] OR "Hospitals"[Mesh] OR "Intensive Care Units"[Mesh] OR "Nursing Homes"[Mesh] OR hospital*[Title/Abstract] OR healthcare[Title/Abstract] OR "health care"[Title/Abstract] OR "healthcare setting*"[Title/Abstract] OR "health care setting*"[Title/Abstract] OR ICU[Title/Abstract] OR "intensive care"[Title/Abstract] OR "long-term care"[Title/Abstract] OR "skilled nursing facilit*"[Title/Abstract] OR "nursing home*"[Title/Abstract])) | 222 |
| Web of Science Core Collection | TS=("*Candida auris*" OR "*C. auris*" OR "*Candidozyma auris*") AND TS=("environmental sampling" OR "environmental surveillance" OR "environmental culture*" OR "environmental swab*" OR "surface sampling" OR "surface culture*" OR "surface swab*" OR "environmental contamination" OR "surface contamination" OR "healthcare environment*" OR "health care environment*" OR "hospital environment*" OR fomite* OR (environment* NEAR/3 (surface* OR equipment OR device* OR "medical equipment" OR "reusable equipment" OR "patient room*"))) AND TS=(hospital* OR healthcare OR "health care" OR "healthcare setting*" OR "health care setting*" OR ICU OR "intensive care" OR "long-term care" OR "skilled nursing facilit*" OR "nursing home*") | 208 |
| Elsevier Embase | ('*candida auris*'/exp OR '*Candida auris*':ti,ab,kw OR '*C. auris*':ti,ab,kw OR '*Candidozyma auris*':ti,ab,kw) AND ('environmental monitoring'/exp OR 'environmental sampling':ti,ab,kw OR 'environmental surveillance':ti,ab,kw OR 'environmental culture*':ti,ab,kw OR 'environmental swab*':ti,ab,kw OR 'surface sampling':ti,ab,kw OR 'surface culture*':ti,ab,kw OR 'surface swab*':ti,ab,kw OR 'environmental contamination':ti,ab,kw OR 'surface contamination':ti,ab,kw OR 'healthcare environment*':ti,ab,kw OR 'health care environment*':ti,ab,kw OR 'hospital environment*':ti,ab,kw OR fomite*:ti,ab,kw OR ('environment*' NEAR/3 ('surface*' OR equipment OR device* OR 'medical equipment' OR 'reusable equipment' OR 'patient room*')):ti,ab,kw) AND (hospital*:ti,ab,kw OR healthcare:ti,ab,kw OR 'health care':ti,ab,kw OR 'healthcare setting*':ti,ab,kw OR 'health care setting*':ti,ab,kw OR ICU:ti,ab,kw OR 'intensive care':ti,ab,kw OR 'long-term care':ti,ab,kw OR 'skilled nursing facilit*':ti,ab,kw OR 'nursing home*':ti,ab,kw) | 218 |
| Scopus | TITLE-ABS-KEY("*Candida auris*" OR "*C. auris*" OR "*Candidozyma auris*") AND TITLE-ABS-KEY("environmental sampling" OR "environmental surveillance" OR "environmental culture*" OR "environmental swab*" OR "surface sampling" OR "surface culture*" OR "surface swab*" OR "environmental contamination" OR "surface contamination" OR "healthcare environment*" OR "health care environment*" OR "hospital environment*" OR fomite* OR (environment* W/3 (surface* OR equipment OR device* OR "medical equipment" OR "reusable equipment" OR "patient room*"))) AND TITLE-ABS-KEY(hospital* OR healthcare OR "health care" OR "healthcare setting*" OR "health care setting*" OR ICU OR "intensive care" OR "long-term care" OR "skilled nursing facilit*" OR "nursing home*") | 244 |

Note: ICU, intensive care unit. The final search was conducted on July 5, 2026. Records retrieved are database-specific counts before duplicate removal. Search strings were adapted to the syntax of each database. An asterisk (*) indicates truncation or wildcard searching. NEAR/3 and W/3 indicate proximity operators requiring terms to appear within three words of each other. Searches covered each database from inception to July 5, 2026. No language restrictions were applied at the search or study-selection stages. No separate gray-literature or institutional-website search was conducted.
